# Angiotensin II Promotes Progressive Activation of Fibrogenic Periostin-Lineage Cells in Lung and Kidney

**DOI:** 10.3390/cells14201584

**Published:** 2025-10-11

**Authors:** Mustafa Ozdemir, José P. Guirao-Abad, Daniel A. Kasprovic, Robert M. Jaggers, Onur Kanisicak

**Affiliations:** 1 Pathobiology and Molecular Medicine Program, Department of Pathology and Laboratory Medicine, University of Cincinnati College of Medicine, Cincinnati, OH 45267, USA; 2 Division of Basic and Translational Science, Department of Emergency Medicine, The Ohio State University Wexner Medical Center, Columbus, OH 43210, USA; jose.guiraoabad@osumc.edu (J.P.G.-A.); kasprovic.2@osu.edu (D.A.K.); robert.jaggers@osumc.edu (R.M.J.); 3 Dorothy M. Davis Heart and Lung Research Institute, The Ohio State University Wexner Medical Center, Columbus, OH 43210, USA

**Keywords:** angiotensin II, fibroblast, periostin-lineage, tissue remodeling, Postn, AngII

## Abstract

Angiotensin II (AngII), the primary effector of the renin-angiotensin system, is essential for maintaining blood pressure and fluid-electrolyte homeostasis. However, elevated AngII levels are a feature of disease conditions such as heart failure and chronic kidney disease, where it is associated with pathological tissue remodeling and fibrosis. AngII-mediated fibrosis has been documented in multiple organs and is characterized by fibroblast expansion, myofibroblast differentiation, and excessive extracellular matrix deposition. Periostin has recently emerged as a marker of fibroblast activation. Notably, periostin expression is highly upregulated during fibrotic remodeling in the kidney and lung, which is strongly linked with impaired organ function. While AngII-induced activation of periostin-lineage (Postn^Lin^) cells is well established in the heart, the temporal dynamics of Postn^Lin^ activation in response to AngII infusion in the lung and kidney remain unexplored. Here, we used a Postn-MerCreMer lineage-tracing approach, combined with continuous AngII infusion over an experimental period of one week and two weeks to assess Postn^Lin^ responses in lung and kidney. Our findings reveal a progressive activation of Postn^Lin^ cells in both organs, characterized by myofibroblast phenotype, together with increased collagen deposition and macrophage infiltration. These results highlight the potential of Postn^Lin^ fibroblasts as a key effector of AngII-mediated tissue remodeling and fibrosis in the lung and kidney.

## 1. Introduction

Angiotensin II (AngII), the primary effector of the renin-angiotensin system (RAS), plays a central role in regulating blood pressure and maintaining fluid-electrolyte balance [1]. Beyond its physiological role in homeostasis, sustained elevated AngII levels are a feature of several chronic diseases. For example, patients with chronic heart failure or chronic kidney disease often exhibit plasma AngII concentrations markedly higher than those in healthy individuals [1,2]. Thus, increased AngII levels are closely associated with pathological tissue remodeling and fibrosis, processes that compromise organ function and, in severe cases, result in organ failure. AngII-mediated fibrotic remodeling has been documented across multiple organs, including the heart, lung, kidney, and skeletal muscle [1,3,4,5,6,7]. The key features of this pathological process include the expansion and accumulation of fibroblast populations, their differentiation into myofibroblast (activated fibroblasts), and the subsequent deposition of excessive extracellular matrix (ECM) accompanied by increased collagen synthesis [1,3,8].

Fibroblasts are traditionally recognized for their supportive role in maintaining tissue structure through the synthesis and remodeling of ECM. However, growing evidence reveals that fibroblasts constitute a highly heterogeneous cell population with diverse and dynamic roles not only in the preservation of tissue homeostasis but also in contributing to pathological processes and disease progression [9,10,11,12]. Particularly, a fibroblast adopts a transient and contractile myofibroblast phenotype and acts as a signaling cue to mediate the surrounding cellular niche in response to tissue damage. Also, AngII-activated fibroblasts typically adopt a myofibroblast phenotype, a specialized cell type with pronounced contractile properties and enhanced ECM production, defined by the expression of molecular markers such as alpha-smooth muscle actin (αSMA) and vimentin. Accordingly, myofibroblast activation is widely recognized as a hallmark of fibrosis development [3,13,14]. In lungs and kidneys, AngII can stimulate fibroblast proliferation, induce their transition into myofibroblasts and enhance ECM remodeling and collagen production [1,7,15,16].

Recently, periostin has emerged as a selective and comprehensive marker of activated fibroblasts [5,17]. Periostin is a secreted matricellular protein that is robustly upregulated in response to injury, while most resident fibroblasts in adult tissue exhibit minimal or no expression profile under homeostatic conditions [5]. In the heart, AngII is known to stimulate periostin-lineage (Postn^Lin^) cell activation and upregulate periostin expression, together with induction of activated fibroblast phenotype [5,18,19]. In addition, periostin expression is upregulated during fibrotic remodeling in the kidney [20,21,22] and lung [23,24] which is strongly linked with impaired organ function. Despite these observations, the temporal dynamics and phenotypic characteristics of Postn^Lin^ cells in the lung and kidney in response to AngII infusion remain unexplored. To address this gap, we employed a previously established Periostin-MerCreMer lineage-tracing model in combination with continuous AngII infusion over a two-week experimental period in mice. Our findings demonstrate that AngII infusion induces a progressive activation of Postn^Lin^ cells in both lung and kidney, characterized by a myofibroblast phenotype accompanied by increased collagen deposition and macrophage infiltration.

## 2. Materials and Methods

### 2.1. Animals and Institutional Approval

All animal procedures in this study were approved by the Institutional Animal Care and Use Committee of the Ohio State University. The experimental mice were cared for in accordance with the guidelines provided for the Care and Use of Laboratory Animals. Mice were maintained under a standard 12:12 h light/dark cycle, with unrestricted access to food and water.

### 2.2. Mouse Lines

We employed the following transgenic mouse line in our experiments: Rosa26-tdTomato (B6. Cg-Gt(rosa)26Sortm14(CAG-tdTomato)Hze/J; JAX Strain#: 007914), and Pdgfrα-eGFP (B6.129S4-Pdgfrαtm11(EGFP)Sor/J; JAX Strain #: 007669) mice that were purchased from the Jackson Laboratory (Bar Harbor, ME, USA). Postn-MerCreMer (PostnMCM) (B6.129S post-ntm2.1(cre/Esr1*) Jmol/J; JAX Strain: 029645) mice were generated as previously described [5,25]. Briefly, lineage tracing was employed by crossing Postn-MerCreMer mice with Rosa26-tdTomato reporter mice, enabling tamoxifen (TAM)-inducible Cre-mediated activation of tdTomato expression in Postn-expressing cells.

### 2.3. Tamoxifen (TAM) Preparation and Administration

Tamoxifen (TAM; AdipoGen, San Diego, CA, USA 50-149-0595) was freshly prepared at 20 mg/mL in 10% ethanol and 90% sunflower oil. Experimental mice received a single oral gavage dose of 5 mg of TAM per 30 g of body weight on the day of pump implantation. To maintain sustained Cre recombination, tamoxifen citrate-infused chow (400 mg/kg; Inotiv, West Lafayette, IN, USA TD130860) was provided ad libitum throughout the experimental period.

### 2.4. Angiotensin II Pump Implantation

Continuous angiotensin II (AngII; Sigma, St. Louis, MO, USA A9525-50G) and phenylephrine hydrochloride (PE; Sigma, P6126) were administered using Alzet micro-osmotic pumps (Alzet; Model 1002, Durect Corporation, Cupertino, CA, USA). Micro-osmotic pumps were prepared to deliver the combination dose of AngII at 1.5 µg/g/d and PE at 50 µg/g/d. While the mice were under light isoflurane sedation, the dorsal mid-scapular region was shaved and disinfected. About a 1 cm incision was performed, and a subcutaneous pocket was created by blunt dissection. Each micro-pump was subcutaneously inserted into the incision. Following pump implantation, the incision was closed with wound clips, and mice were allowed to recover on a warming pad. Mice were monitored during recovery, and postoperative analgesia (buprenorphine, 0.1 mg/kg, subcutaneously) was provided by institutional animal care protocols. Sham control mice received identical surgical procedures with a micro-pump containing sterile saline instead of AngII. All procedures were performed under aseptic conditions.

### 2.5. Tissue Harvest

Mice were euthanized at designated experimental endpoints of 7 or 14 days post-pump implantation. The lung and kidney were promptly dissected. Freshly isolated organs were immediately immersed in freshly prepared 4% paraformaldehyde (PFA) for 2 h at room temperature to ensure optimal fixation. Following fixation, tissues were rinsed thoroughly with phosphate-buffered saline (PBS) and transferred into 30% sucrose in PBS overnight at 4 °C. The following day, tissues were embedded in optimal cutting temperature (OCT) compound (Tissue Plus, Thermo Fisher Scientific, Waltham, MA, USA) and stored at −80 °C until further analysis.

### 2.6. Histology and Immunohistochemistry

Frozen tissues embedded in OCT were sectioned at 10 µm thickness using a cryostat (Leica CM1950, Leica Biosystem, Wetzlar, Germany) and adhered to multiple positively charged microscope slides (Fisher Scientific, 1255015); then slides were allowed to air dry for 10 min prior to being stored at −80 °C until further analysis. Slides were next incubated for 1 h at room temperature in a freshly prepared blocking solution containing 5% goat serum, 2% bovine serum albumin (BSA), and 0.1% Triton X-100 in PBS. The same solution was used for primary and secondary antibody dilutions. Following blocking, sections were incubated overnight at 4 °C with primary antibody (1:100 or 1:200 dilutions). The next day, slides were washed with PBS three times for 5 min each. Then, sections were incubated for 1 h at room temperature with secondary antibody (1:500 dilution). After three additional PBS washes of 5 min each, slides were mounted with an aqueous mounting medium containing DAPI (Vector Laboratories, H1200, Burlingame, CA, USA) to counterstain the nuclei and mounted with microscope glass cover slips (Fisher Scientific, 12545E). The primary antibodies used in this study were as follows: rat anti-CD31 (BD Biosciences, San Jose, CA, USA 553370), rat anti-CD45 (BD Biosciences, San Jose, CA, USA 553070), rat anti-CD68 (BioLegend, San Diego, CA, USA 137002), mouse anti-αSMA conjugated to Alexa Fluor 488 (AF488; eBioscience, Thermo Fisher Scientific, Waltham, MA, USA 53-9760-82), rabbit anti-vimentin conjugated to AF488 (Cell Signaling Technology, Danvers, MA, USA 9854), and rabbit anti-col1a1 (Cell Signaling Technology, Danvers, MA, USA, 72026). Secondary antibodies included goat anti-rabbit IgG AF488 (Abcam, Waltham, MA, USA ab150077), goat anti-rat AF647 (Invitrogen, Thermo Fisher Scientific, Waltham, MA, USA A21247), and goat anti-rabbit AF647 (Invitrogen, Thermo Fisher Scientific, Waltham, MA, USA A21245). Cryosections were then used to visualize the native fluorescent reporter as well as antibody-labeled target proteins. Notably, secondary-only sections were included to validate antibody specificity; no signal above background was detected.

Digital images were captured using a Nikon AXR confocal microscope (Nikon Instruments Inc., Tokyo, Japan). Subsequent analysis was performed using Fiji (ImageJ version 1.53t; (National Institutes of Health, Bethesda, MD, USA).

### 2.7. Flow Cytometry Analysis

Harvested hearts were rinsed in cold PBS and transferred to C-tubes (Miltenyi Biotec, Bergisch Gladbach, Germany 130-093-237) containing 5 mL of digestion buffer composed of Dulbecco’s PBS supplemented with 0.9 mM CaCl_2_, 600 U/mL Collagenase IV (Worthington Biochemical Corporation, Lakewood, NJ, USA, LS004189), 1.2 U/mL Dispase II (Gibco, Thermo Fisher Scientific, Waltham, MA, USA 17105041), and 30 U/mL DNase I (Sigma, D4527-10KU). Tissue samples were incubated at 37 °C for 35 min in a water bath and subsequently dissociated using a MACS™ Dissociator (Miltenyi Biotec, 130-093-235). The resulting homogenate was passed through a 40 µm cell strainer. Red blood cells were removed by treatment with Red Cell Lysis Buffer (eBioscience, 00-4300-54) according to the manufacturer’s protocol. The remaining cell suspension was washed and resuspended in fluorescence-activated cell sorting (FACS) buffer containing DPBS (Gibco, 14190-136) with 0.09% sodium azide and 1% FBS. Peripheral blood was collected into EDTA K3E tubes (Sarstedt, Newton, NC, USA 20.1341) and subjected to red blood cell lysis using RBC Lysis Buffer according to the manufacturer’s protocol. Following lysis, samples were washed three times and resuspended in FACS buffer. Spectral flow cytometry analysis was performed using a Cytek Bioscience Aurora Spectral Analyzer with the following five laser configurations: 355, 405, 488, 561, and 640 nm, and 64 detection channels to quantify Postn tdTomoato+ reporter cells. Samples were gated for debris and doublets before analysis of target populations. Flow cytometry data was generated using FlowJo v.X.X software (BD Biosciences).

### 2.8. Statistical Analysis

Statistical data analysis was performed using GraphPad Prism v10.3.1. All quantitative data are presented as mean ± standard error of the mean (SEM). Comparisons were performed by using a one-way analysis of variance (ANOVA), followed by Tukey’s post hoc test where appropriate. A *p*-value of <0.05 was considered statistically significant.

## 3. Results

### 3.1. AngII Infusion Progressively Activates Postn^Lin^ Cells Accompanied by Increased Macrophage Infiltration and Collagen Deposition

**Figure 1:**
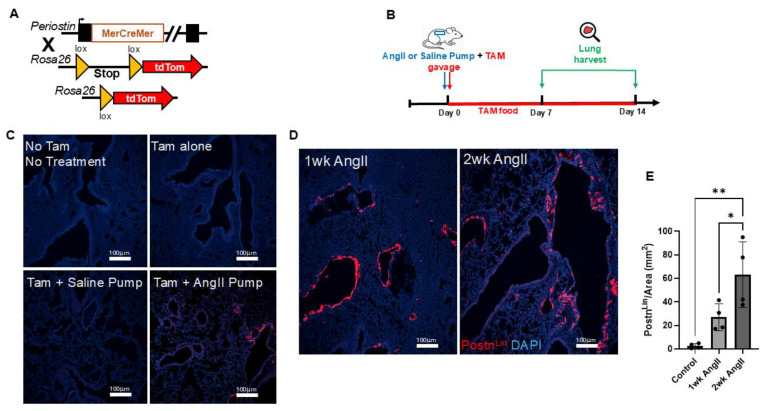
Experimental design and periostin-lineage cell activation in lung following angiotensin II (AngII) infusion. (**A**) Schematic representation of the Postn genetic locus with a tamoxifen-regulated MerCreMer crossing with Rosa26-tdTomato reporter mice. (**B**) Experimental design showing that tamoxifen (TAM) was administered via oral gavage on the day of either AngII or saline mini-pump implantation. Lungs were harvested on days 7 and 14 post-treatment. (**C**) Representative lung images showing baseline and control conditions. (**D**) Representative lung sections from mice treated with AngII for 1 week and 2 weeks show progressive activation and expansion of tdTomato^+^ periostin-lineage cells. (**E**) Quantification of periostin-lineage (tdTomato^+^) cells per mm^2^ in lung tissue reveals a significant and progressive increase following 1 and 2 weeks of AngII treatment compared to saline pump control (*n* = 4 per group). For each sample, at least four 10× images from distinct regions and depths of the lung were quantified. Data are presented as mean ± SEM. (* = *p* < 0.05; ** = *p* < 0.01, one-way ANOVA with Tukey’s post hoc test). tdTomato⁺ periostin-lineage cells are shown in red, and nuclei (DAPI) are shown in blue. Scale bar = 100 µm.

To track the activation of the Postn promoter, one allele of the Postn gene was replaced via targeted insertion using a TAM-inducible Cre recombinase with Postn-MerCreMer mice. This mouse line was then crossed with a reporter strain that permanently expresses tdTomato fluorescence in response to Cre activity (Figure 1A). These Postn^Lin^-tracing mice were administered TAM via oral gavage on the day of osmotic mini-pump implantation, and mice were maintained on TAM chow throughout the experimental period to ensure continuous Cre activity (Figure 1B). As expected, no tdTomato fluorescence was detected in the lungs of Postn^Lin^ mice receiving TAM alone or untreated controls without TAM and pump (Figure 1C). In the lungs of Postn^Lin^ mice given TAM together with saline pump implantation, only sporadic tdTomato+ fluorescence was observed, confirming tight control of reporter gene expression in the absence of Cre-mediated recombination (Figure 1C). In contrast, robust Postn-tdTomato expression emerged in the lungs of mice treated with an AngII pump together with TAM (Figure 1C). Furthermore, Postn^Lin^-tracing mice demonstrated that continuous AngII infusion for two weeks induced a marked, time-dependent activation and expansion of Postn^Lin^ cells in mice lungs (Figure 1D). Indeed, quantitative analysis revealed a progressive increase in activated Postn^Lin^ cells over the treatment period, indicating sustained Postn^Lin^ activation in response to AngII infusion (Figure 1E).

**Figure 2:**
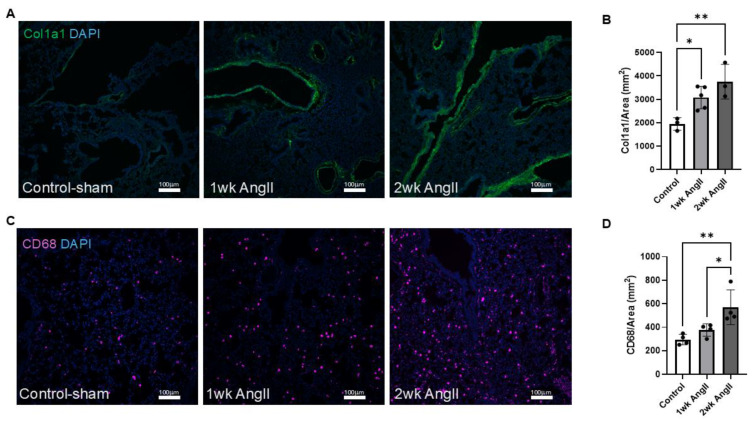
AngII induces progressive collagen deposition and macrophage infiltration in the lung. (**A**) Representative immunofluorescent images showing col1a1 expression in lung tissue from control-sham (*n* = 3), 1-week AngII pump (*n* = 5), and 2-week AngII (*n* = 3) pump-treated mice. (**B**) Quantification of col1a1 mean gray value normalized to tissue area (mm^2^) reveals a significant and progressive increase in collagen deposition following 1 and 2 weeks of AngII treatment compared to control. (**C**) Representative images of CD68 staining in lung sections from control-sham, 1-week, and 2-week AngII groups, demonstrating increased macrophage infiltration. (**D**) Quantification of CD68^+^ cells per mm^2^ shows a time-dependent rise in macrophage content following AngII administration (*n* = 4 per group). For each sample, at least four 10× (for col1a1) or 20× (for CD68) images from distinct regions and depths of the lung were quantified. Data are presented as mean ± SEM. (* = *p* < 0.05; ** = *p* < 0.01, one-way ANOVA with Tukey’s post hoc test). Col1a1 is shown in green, CD68 in magenta, and nuclei (DAPI) in blue. Scale bar = 100 µm.

Next, the lung sections were also examined for collagen deposition and macrophage infiltration. As mentioned previously, collagen deposition is a hallmark feature of fibrotic tissue remolding. Thus, AngII can promote collagen deposition both in the lung and kidney. Here, we employed col1a1 immunofluorescence staining to assess collagen production in the lungs following AngII infusion. Our results revealed a progressive increase in collagen deposition over time, with levels becoming more pronounced as the treatment continued (Figure 2A,B). Although our analysis reflects the entire lung section for col1a1 staining, prior studies have shown that AngII induces collagen deposition in both parenchymal and perivascular compartments [7,26,27]. Macrophage presence was identified in lung tissue using immunofluorescence staining for CD68, a well-established myeloid cell marker that is abundantly expressed by macrophages [28]. Evidence indicates that AngII-mediated macrophage recruitment is involved in fibrotic remodeling of both lung and kidney tissues [1,7,29]. In this study, immunohistochemical analysis of CD68+ cells of lung sections from control (saline sham), 1-week AngII-infused, and 2-week AngII-infused mice showed a progressive increase in CD68^++^ cells (Figure 2C,D). Collectively, consistent with previous reports, these findings demonstrate that AngII infusion promotes progressive macrophage recruitment to the lung, as reflected by increased CD68^+^ cell abundance, together with increased collagen production.

### 3.2. AngII Infusion Stimulated Postn^Lin^ Cells Exhibit Activated Fibroblast Phenotype in the Lung

**Figure 3:**
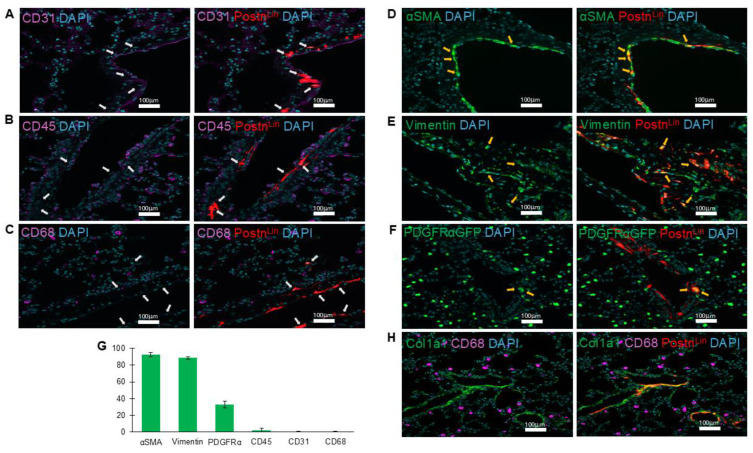
Periostin-lineage cells colocalize with fibrogenic but not immune or endothelial markers in the lung following AngII treatment. (**A**–**C**) Representative immunofluorescent images showing lack of co-labeling (white arrows) between periostin-lineage (tdTomato^+^) cells and cellular markers of endothelial (CD31, (**A**)), hematopoietic (CD45, (**B**)), and macrophage (CD68, (**C**)), indicating that activated Postn^Lin^ in response to AngII infusion do not originate from these cell types (*n* = 3–4 per marker). (**D**–**F**) Periostin-lineage cells show colocalization with fibroblast markers: α-SMA (**D**), vimentin (**E**), and PDGFRα-GFP (**F**), suggesting an activated fibroblast phenotype (yellow arrows indicate areas of co-labeling). (**G**) Quantification of co-labeling of tdTom^+^ (periostin^+^) cells with indicated cell markers from immunohistochemically processed lung sections of AngII pump-implanted mice. Data represents an average of three to four lungs per marker, with >5 sections quantified from each. Data are presented as mean ± SEM. (**H**) Representative immunofluorescent image of collagen type 1 (col1a1), macrophages (CD68), and Postn^Lin^ cells. Images are representative of lungs harvested after AngII treatment (*n* = 3/4 per marker). tdTomato⁺ periostin-lineage cells are shown in red; nuclei (DAPI) in blue; CD31, CD45, and CD68 in magenta; and α-SMA, vimentin, PDGFRα-GFP, and Col1a1 in green. Scale bar = 100 µm.

Pathological tissue remodeling is characterized by proliferation and accumulation of fibroblast cell populations, their differentiation into myofibroblasts (activated fibroblasts), and subsequent deposition of excessive ECM. Notably, AngII has been shown to promote each of these processes in the lung [1,7]. Here, to define the phenotypic characteristics of Postn^Lin^ cell activation in response to AngII infusion, we performed immunofluorescence staining for established markers of activated fibroblasts, including αSMA and vimentin. In parallel, we assessed additional cell populations using CD31, CD45, and CD68 cell markers to identify endothelial cells, hematopoietic cells, and macrophages, respectively. Our analysis revealed that AngII-activated Postn^Lin^ cells did not belong to endothelial cells, hematopoietic cells, and macrophage populations (CD31-, CD45-, and CD68-, respectively) (Figure 3A–C). By contrast, immunofluorescence examinations revealed that AngII-induced activated Postn^Lin^ cells often exhibited co-staining with αSMA and vimentin (Figure 3D,E), markers that are commonly used to label activated fibroblasts.

Platelet-derived growth factor α (PDGFRα) is widely recognized as a marker of tissue-resident fibroblasts across multiple organs, including the lung [12,30]. To assess whether Postn^Lin^ cells induced by AngII infusion co-express PDGFRα, we utilized the Postn^Lin^/PDGFRα-GFP mouse strain. Examination of lung sections following AngII infusion revealed a subset of tdTomato+ cells co-expressing PDGFRα-GFP (Figure 3F). Overall, the quantification analysis of co-labeling of periostin^+^ cells showed ~90–95% co-staining with αSMA and vimentin and ~35% co-localization with PDGFRα-GFP, while minimal or no co-labeling was detected with other markers (Figure 3G). This observation aligns with previous reports indicating >95% co-staining with α-SMA and vimentin and up to ~60% Postn^Lin^ cells co-express PDGFRα in both the heart and lung in response to injury or infection [5,25]. Notably, immunohistochemical images revealed enriched clusters of Postn^Lin^ cells co-localizing with collagen, whereas macrophages showed no evidence of co-labeling but were frequently observed in close proximity (Figure 3H). Collectively, we conclude that AngII infusion induces progressive activation of Postn^Lin^ cells in the lung, which exhibit phenotypic characteristics of myofibroblasts.

### 3.3. AngII Infusion Progressively Activates Postn^Lin^ Cells Accompanied by Increased Macrophage Infiltration and Collagen Deposition in the Kidney

**Figure 4:**
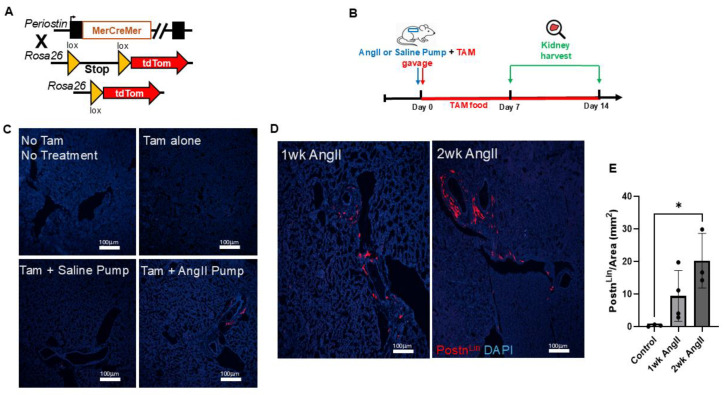
Experimental design and periostin-lineage cell activation in kidney following AngII infusion. (**A**) Schematic representation of the Postn genetic locus with a tamoxifen-regulated MerCreMer crossing with Rosa26-tdTomato reporter mice. (**B**) Experimental design showing that tamoxifen (TAM) was administered via oral gavage on the day of either AngII or saline mini-pump implantation. Kidney was harvested on days 7 and 14 post-treatment. (**C**) Representative kidney images show baseline and control conditions. (**D**) Representative kidney sections from mice treated with AngII for 1 week and 2 weeks show progressive activation and expansion of tdTomato^+^ periostin-lineage cells. (**E**) Quantification of periostin-lineage (tdTomato^+^) cells per mm^2^ in kidney tissue reveals a significant and progressive increase following 1 and 2 weeks of AngII treatment compared to saline pump control (*n* = 3–4 per group). For each sample, at least four 10× images from distinct regions and depths of the kidney were quantified. Data are presented as mean ± SEM. (* = *p* < 0.05, one-way ANOVA with Tukey’s post hoc test). tdTomato⁺ periostin-lineage cells are shown in red, and nuclei (DAPI) are shown in blue. Scale bar = 100 µm.

To determine if the Postn^Lin^ cells in the kidney respond similarly to AngII infusion as observed in the lung, we performed lineage-tracing experiments using Postn-MerCreMer; Rosa26tdTomato reporter mice (Figure 4A). As explained in the previous section, lineage-tracing mice were administered TAM via oral gavage on the same day as osmotic mini-pump implantation, and mice were maintained on TAM chow throughout the experimental period to ensure continuous Cre activity (Figure 4B). Kidneys were harvested at designated time points of 7 days and 14 days post either AngII or saline pump implementation (Figure 4B). Kidneys from no-treatment or TAM-only control mice displayed no detectable tdTomato fluorescence signal (Figure 4C). Similarly to lungs, only sporadic tdTomato^+^ cells were observed in saline-pump-implanted (control) mice, again confirming the tight regulation of reporter activation in the absence of Cre-mediated recombination (Figure 4C). In contrast, robust and progressive activation of Postn^Lin^ cells emerged in kidney sections of AngII pump-implanted mice (Figure 4D); indeed, quantitative analysis revealed a time-dependent increase in Postn^Lin^ cells in the kidney (Figure 4E), mirroring the progressive expansion seen in the lung.

**Figure 5:**
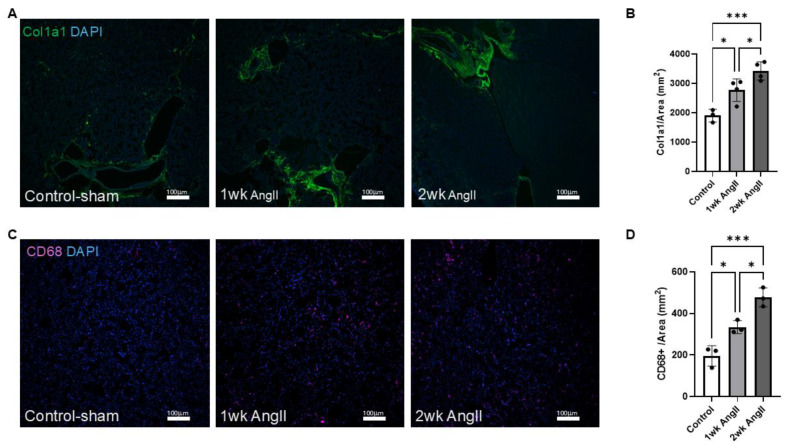
AngII induces progressive collagen deposition and macrophage infiltration in the kidney. (**A**) Representative immunofluorescent images showing col1a1 expression in kidney tissue from control-sham, 1-week AngII pump, and 2-week AngII pump-treated mice. (**B**) Quantification of col1a1 mean gray value normalized to tissue area (mm^2^) reveals a significant and progressive increase in collagen deposition following 1 and 2 weeks of AngII treatment compared to control (*n* = 3–4 per group). (**C**) Representative images of CD68 staining in kidney sections from control-sham, 1-week, and 2-week AngII groups, demonstrating increased macrophage infiltration. (**D**) Quantification of CD68^+^ cells per mm^2^ shows a time-dependent rise in macrophage content following AngII administration (*n* = 3 per group). For each sample, at least four 10× (for col1a1) or 20× (for CD68) images from distinct regions and depths of the kidney were quantified. Data are presented as mean ± SEM. (* = *p* < 0.05; *** = *p* < 0.001, one-way ANOVA with Tukey’s post hoc test). Col1a1 is shown in green, CD68 in magenta, and nuclei (DAPI) in blue. Scale bar = 100 µm.

Next, we evaluated kidney sections to determine the collagen production and macrophage infiltration in response to AngII infusion. Similarly to observations seen in the lungs, immunofluorescence staining of col1a1 indicated a progressive increase in collagen production in the kidneys of AngII-infused mice (Figure 5A,B). Additionally, macrophages in the kidney were identified by immunofluorescence staining with the CD68 marker (Figure 5C). The analysis indicates a progressive increase in macrophage recruitment over time in response to AngII infusion, which was further confirmed by quantitative analysis (Figure 5D).

### 3.4. AngII Infusion Stimulated Postn^Lin^ Cells Exhibit Activated Fibroblast Phenotype in the Kidney

**Figure 6:**
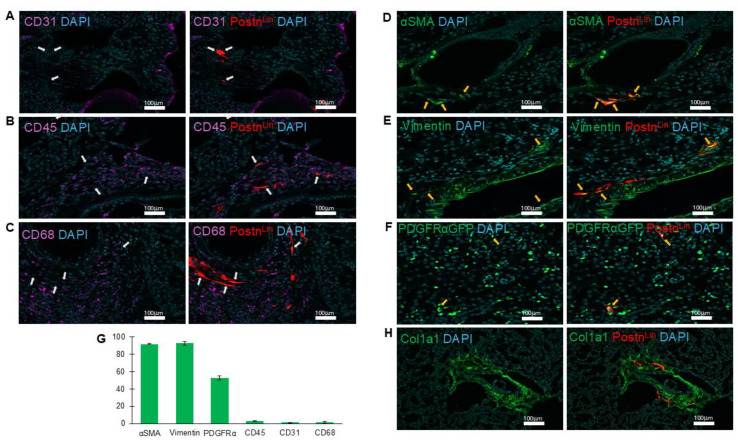
Periostin-lineage cells colocalize with fibrogenic but not immune or endothelial markers in the kidney following AngII treatment. (**A**–**C**) Representative immunofluorescent images showing lack of co-labeling (white arrows) between periostin-lineage (tdTomato^+^) cells and cellular markers of endothelial (CD31, (**A**)), hematopoietic (CD45, (**B**)), and macrophage (CD68, (**C**)), indicating activated Postn^Lin^ in response to AngII infusion do not originate from these cell types (*n* = 3/4 per marker). (**D**–**F**) Periostin-lineage cells indicate co-staining with fibroblast markers: α-SMA (**D**), vimentin (**E**), and PDGFRα-GFP (**F**), suggesting an activated fibroblast phenotype (yellow arrows indicate areas of co-labeling). (**G**) Quantification of co-labeling of tdTom^+^ (periostin^+^) cells with indicated cell markers from immunohistochemically processed kidney sections of AngII pump-implanted mice. Data represents an average of three to four kidneys per marker, with >5 sections quantified from each. Data are presented as mean ± SEM. (**H**) Representative immunofluorescent image of collagen type 1 (col1a1) and Postn^Lin^ cells. Images are representative of kidneys harvested after AngII treatment (*n* = 3/4 per marker). tdTomato⁺ periostin-lineage cells are shown in red; nuclei (DAPI) in blue; CD31, CD45, and CD68 in magenta; and α-SMA, vimentin, PDGFRα-GFP, and Col1a1 in green. Scale bar = 100 µm.

To determine the phenotypic characteristics of activated Postn^Lin^ cells in the kidney, we performed immunofluorescence staining of αSMA and vimentin, with the use of additional markers to identify endothelial cells (CD31), hematopoietic cells (CD45), and macrophages (CD68). Our analysis revealed that AngII-activated Postn^Lin^ cells in the kidney did not belong to endothelial, hematopoietic, or macrophage populations (CD31-, CD45-, CD68-, respectively) (Figure 6A–C). By contrast, immunofluorescence analysis demonstrated that AngII-induced activated Postn^Lin^ cells in the kidney often co-express αSMA, vimentin, and PDGFRα-GFP (Figure 6D–F). In addition, the quantification analysis of co-labeling with periostin^+^ cells showed >90% co-staining with α-SMA and vimentin and ~50% co-labeling with PDGFRα-GFP, while minimal or no co-labeling was detected with other markers on kidney sections (Figure 6G). Similarly to the lung, enriched clusters of Postn^Lin^ cells were also observed in the kidney, where they co-localized with collagen deposition (Figure 6H). Collectively, these results indicate that AngII-activated Postn^Lin^ cells in the kidney exhibit an activated fibroblast phenotype.

### 3.5. Flow Cytometry Analysis Indicates Postn^Lin^ Cells Are Locally Produced and Absent from Circulation

The flow cytometry analysis was performed on freshly isolated heart tissue and peripheral blood samples of a wild type and Postn^Lin^ mice implanted with an AngII pump in representative proof-of-concept experiments. The analysis identified a substantial population of tdTomato^+^ periostin-lineage cells within the heart, whereas no detectable tdTomato^+^ cells were observed in the peripheral blood (Supplementary Figure S1).

## 4. Discussion

In this study, we demonstrate that AngII infusion drives progressive activation of Postn^Lin^ cells in both lungs and kidneys, accompanied by hallmark features of pathological remodeling. Briefly, to investigate the effects of AngII infusion on lung and kidney tissue, we employed a common preclinical model that mimics the disease conditions associated with elevated AngII levels in humans, combined with a novel lineage-tracing approach. Importantly, our results provide new and novel insights into the contribution of AngII-mediated Postn^Lin^ cell activation in lung and kidney remodeling. Moreover, our data show that AngII-activated Postn^Lin^ cells are distinct from endothelial, hematopoietic, and macrophage populations but instead co-express classical markers of activated fibroblasts, including αSMA, vimentin, and resident/activated fibroblast marker PDGFRα. Taken together, these findings firmly establish that Postn^Lin^ cells are a key fibroblast-derived population activated in response to AngII in both lungs and kidneys. Consistent with this activation, AngII infusion was associated with progressive collagen deposition, as evidenced by increased collagen (col1a1) staining, and with enhanced macrophage infiltration, reflected by elevated CD68^+^ cell abundance. Collectively, these findings identify Postn^Lin^ cells as an important fibrogenic population in AngII-mediated remodeling of lung and kidney and highlight their close spatial relationship with immune infiltration and collagen deposition during AngII-mediated response.

Our previous work demonstrated that Postn^Lin^ cells are a major source of collagen that are distinct from macrophage populations in injured hearts [5]. In line with this, our current analysis shows no co-localization between Postn^Lin^ cells and the macrophage marker CD68. Moreover, recent studies have reinforced that resident macrophages and infiltrating monocytes do not transdifferentiate into fibroblasts or myofibroblasts, nor do they directly contribute to structural collagen deposition in fibrotic tissue [31,32]. Nevertheless, future studies will be important to delineate the roles of macrophages, such as paracrine signaling or modulation of fibroblast activity, in regulating tissue-specific collagen production in response to diverse injuries. While periostin was reported to be induced in macrophages following AngII [33], our co-immunohistochemical staining did not reveal co-localization of the periostin-lineage signal with either CD45 (hematopoietic cells) or CD68 (macrophage) across the sections examined, indicating that the lineage in our model predominantly marks non-hematopoietic stromal cells, consistent with our prior reports that periostin-lineage cells correspond to the fibroblast population [5,25]. Although we observed progressive macrophage accumulation after AngII infusion, the functional phenotype of these cells was not investigated. Future studies employing subset-specific immunophenotyping and cytokine/chemokine profiling will be essential to determine whether inflammatory versus alternatively activated macrophages differentially interact with fibroblast populations in response to AngII.

Systemic AngII infusion using osmotic mini pumps is a well-established and widely used preclinical model that mimics the conditions of elevated AngII levels observed in humans [34]. While the use of this preclinical model provides valuable insight into AngII-driven mechanisms that compromise tissue integrity and function, several limitations should be addressed. Although AngII infusion via pumps effectively elevates circulating AngII levels, it remains uncertain whether this increase recapitulates the rise in tissue-specific RAS activity observed under human pathophysiology. Moreover, the doses commonly used in preclinical studies far exceed the physiological AngII levels present in human conditions [1,34]. Since many components of RAS are expressed by diverse cell types across multiple organ systems, it is difficult to interpret whether systemic elevation of AngII or locally produced AngII plays the predominant role in driving its pathological effects. Furthermore, AngII infusion can trigger both direct and indirect effects on cells and tissues, making it difficult to clearly distinguish the underlying mechanisms. Therefore, findings obtained from this preclinical model should be interpreted with caution and within the context of these inherent limitations.

One of the major pathological consequences of chronically elevated AngII is the induction of fibrotic remodeling. This process is characterized by inflammation, expansion of fibroblast populations, their differentiation into contractile myofibroblast phenotype, and subsequent excessive deposition of ECM, ultimately leading to organ failure. Indeed, AngII has been shown to promote fibroblast proliferation, myofibroblast differentiation, and ECM deposition in both lung and kidney [1,7,15,16]. In line with these reports, our results demonstrate that systemic circulation of AngII induces a progressive activation of Postn^Lin^ cells both in lung and kidney, which displays a firm myofibroblast phenotype. Previous studies have similarly established that injury-induced Postn^Lin^ cells represent activated fibroblast populations in the lung and heart [5,25]. While we demonstrate that Postn^Lin^ cells co-express fibroblast markers (e.g., αSMA, vimentin), the indices of proliferation or apoptosis were not included in this study. Since Postn^Lin^ cells have been shown to proliferate during fibrosis [17], incorporating proliferation and apoptosis assays into future studies will be important to more precisely define the behavior and fate of Postn^Lin^ cells in response to AngII across multiple organs. Future investigations employing single-cell transcriptomics and spatial profiling will also be critical to further validate the role of Postn^Lin^ cells in AngII-induced fibrotic response and to elucidate their interaction with immune and endothelial compartments. Additionally, aging and metabolic diseases are known to potentiate fibroblast activation [11]; therefore, incorporating aged cohorts and models of comorbid conditions (e.g., diabetes, hypertension) will be important to further define how these systemic factors shape Postn^Lin^ cell activation and fibrogenic response to AngII. Thus, future studies using localized AngII delivery and conditional knockout models in Postn^Lin^ or fibroblast subsets will be critical to define organ-specific responses, minimize systemic confounding, and better model the localized RAS activation observed in human disease.

Transforming growth factor β (TGF-β) signaling is a central regulator of fibroblast activation and phenotype across multiple organs, including the lung and kidney [4,35]. Importantly, AngII is known to stimulate TGF-β signaling in these tissues, thereby initiating downstream fibrotic responses [15,36,37,38]. Although the precise mechanisms through which AngII drives Postn^Lin^ activation were not elucidated in our study, periostin has been implicated in both the upstream regulation and downstream responses of the TGF-β signaling [39]. The crosstalk between periostin and TGF-β is established to be critical for amplifying the downstream signaling pathways in pulmonary fibrosis [40]. In addition, TRPC6 has emerged as a critical mechanosensitive element that integrates a response from both TGF-β and AngII, and its activation is essential for promoting fibroblast to myofibroblast transformation [41]. Importantly, AngII can also induce myofibroblast differentiation through TGF-β-independent mechanisms [41]. Interestingly, evidence also suggests a bidirectional interaction between myofibroblasts and locally produced RAS components, as myofibroblasts isolated from fibrotic human lungs have been shown to produce AngII and other RAS metabolites [42]. Thus, future investigations are warranted to define the mechanistic interactions between AngII signaling, periostin, and TGF-β pathways in regulating Postn^Lin^ activation during fibrotic tissue remodeling.

## 5. Conclusions

Emerging evidence suggests that AngII exerts complex and multiple effects across diverse cell types and organs, ultimately compromising tissue integrity and function. Despite extensive investigations, numerous questions remain unanswered regarding the cellular and molecular mechanisms underlying AngII-mediated pathology. Addressing these gaps requires precise identification of the cellular population and signaling pathways involved in AngII-mediated tissue damage. In this study, using a novel lineage-tracing approach, we aimed to elucidate whether systemic AngII infusion activates Postn^Lin^ cells in the lung and kidney. In summary, our findings clearly demonstrate a progressive activation of Postn^Lin^ cells in both organs, characterized by acquisition of an activated fibroblast phenotype accompanied by hallmark features of pathological remodeling, including enhanced collagen deposition and macrophage infiltration. Together, these results highlight Postn^Lin^ fibroblasts as key effectors in AngII-mediated tissue remodeling and underscore their potential role as key drivers of fibrotic progression in the lung and kidney. Lastly, periostin-expressing stromal cells represent a tractable anti-fibrotic target. Preclinical studies in various organs showed that genetic periostin loss or pharmacological inhibition can attenuate matrix remodeling. Given periostin’s context-dependent roles in healing and repair, future work should prioritize temporary, controlled, tissue-specific interventions to test efficacy and safety in organ-specific AngII-induced remodeling.

## Data Availability

The original contributions presented in this study are included in the article. Further inquiries can be directed at the corresponding authors.

## Supplementary Materials

The following supporting information can be downloaded at: https://www.mdpi.com/article/10.3390/cells14201584/s1, Figure S1. Representative flow cytometry plots of isolated tdTom^+^ cells from blood and heart of AngII-treated wild-type (WT, *n* = 1) mice and Postn^Lin^ (Postn^MCM/+^; R26^tdt/+^) mice (*n* = 1).

## Abbreviations

The following abbreviations are used in this manuscript:

AngII	Angiotensin II
PostnLin	Periostin-lineage
ECM	Extracellular matrix
αSMA	Alpha-smooth muscle actin
TAM	Tamoxifen
OCT	Optimal cutting temperature
BSA	Bovine serum albumin
PFA	Paraformaldehyde
PBS	Phosphate-buffered saline
PDGFRα	Platelet-derived growth factor α
RAS	Renin-angiotensin system
TGF-β	Transforming growth factor β

## References

[1] Forrester S.J., Booz G.W., Sigmund C.D., Coffman T.M., Kawai T., Rizzo V., Scalia R., Eguchi S. (2018). Angiotensin II Signal Transduction: An Update on Mechanisms of Physiology and Pathophysiology. Physiol. Rev..

[2] Roig E., Perez-Villa F., Morales M., Jiménez W., Orús J., Heras M., Sanz G. (2000). Clinical implications of increased plasma angiotensin II despite ACE inhibitor therapy in patients with congestive heart failure. Eur. Heart J..

[3] Davis J., Molkentin J.D. (2014). Myofibroblasts: Trust your heart and let fate decide. J. Mol. Cell. Cardiol..

[4] Murphy A.M., Wong A.L., Bezuhly M. (2015). Modulation of angiotensin II signaling in the prevention of fibrosis. Fibrogenesis Tissue Repair..

[5] Kanisicak O., Khalil H., Ivey M.J., Karch J., Maliken B.D., Correll R.N., Brody M.J., Lin S.C.J., Aronow B.J., Tallquist M.D. (2016). Genetic lineage tracing defines myofibroblast origin and function in the injured heart. Nat. Commun..

[6] Bedair H.S., Karthikeyan T., Quintero A., Li Y., Huard J. (2008). Angiotensin II receptor blockade administered after injury improves muscle regeneration and decreases fibrosis in normal skeletal muscle. Am. J. Sports Med..

[7] Uhal B.D., Li X., Piasecki C.C., Molina-Molina M. (2012). Angiotensin signalling in pulmonary fibrosis. Int. J. Biochem. Cell Biol..

[8] Selman M., King T.E., Pardo A. (2001). Idiopathic pulmonary fibrosis: Prevailing and evolving hypotheses about its pathogenesis and implications for therapy. Ann. Intern. Med..

[9] Gomes R.N., Manuel F., Nascimento D.S. (2021). The bright side of fibroblasts: Molecular signature and regenerative cues in major organs. NPJ Regen. Med..

[10] Lendahl U., Muhl L., Betsholtz C. (2022). Identification, discrimination and heterogeneity of fibroblasts. Nat. Commun..

[11] LeBleu V.S., Neilson E.G. (2020). Origin and functional heterogeneity of fibroblasts. FASEB J..

[12] Plikus M.V., Wang X., Sinha S., Forte E., Thompson S.M., Herzog E.L., Driskell R.R., Rosenthal N., Biernaskie J., Horsley V. (2021). Fibroblasts: Origins, definitions, and functions in health and disease. Cell.

[13] Wang J., Zohar R., McCulloch C.A. (2006). Multiple roles of alpha-smooth muscle actin in mechanotransduction. Exp. Cell Res..

[14] Baum J., Duffy H.S. (2011). Fibroblasts and myofibroblasts: What are we talking about?. J. Cardiovasc. Pharmacol..

[15] Rüster C., Wolf G. (2011). Angiotensin II as a morphogenic cytokine stimulating renal fibrogenesis. J. Am. Soc. Nephrol..

[16] Wolf G., Butzmann U., Wenzel U.O. (2003). The renin-angiotensin system and progression of renal disease: From hemodynamics to cell biology. Nephron Physiol..

[17] Kaur H., Takefuji M., Ngai C.Y., Carvalho J., Bayer J., Wietelmann A., Poetsch A., Hoelper S., Conway S.J., Möllmann H. (2016). Targeted Ablation of Periostin-Expressing Activated Fibroblasts Prevents Adverse Cardiac Remodeling in Mice. Circ. Res..

[18] Wu H., Chen L., Xie J., Li R., Li G.N., Chen Q.H., Zhang X.L., Kang L.N., Xu B. (2016). Periostin expression induced by oxidative stress contributes to myocardial fibrosis in a rat model of high salt-induced hypertension. Mol. Med. Rep..

[19] Liu W., Zi M., Tsui H., Chowdhury S.K., Zeef L., Meng Q.J., Travis M., Prehar S., Berry A., Hanley N.A. (2013). A novel immunomodulator, FTY-720 reverses existing cardiac hypertrophy and fibrosis from pressure overload by targeting NFAT (nuclear factor of activated T-cells) signaling and periostin. Circ. Heart Fail..

[20] Wallace D.P., White C., Savinkova L., Nivens E., Reif G.A., Pinto C.S., Raman A., Parnell S.C., Conway S.J., Fields T.A. (2014). Periostin promotes renal cyst growth and interstitial fibrosis in polycystic kidney disease. Kidney Int..

[21] Mael-Ainin M., Abed A., Conway S.J., Dussaule J.C., Chatziantoniou C. (2014). Inhibition of periostin expression protects against the development of renal inflammation and fibrosis. J. Am. Soc. Nephrol..

[22] Hwang J.H., Yang S.H., Kim Y.C., Kim J.H., An J.N., Moon K.C., Oh Y.K., Park J.Y., Kim D.K., Kim Y.S. (2017). Experimental Inhibition of Periostin Attenuates Kidney Fibrosis. Am. J. Nephrol..

[23] Naik P.K., Bozyk P.D., Bentley J.K., Popova A.P., Birch C.M., Wilke C.A., Fry C.D., White E.S., Sisson T.H., Tayob N. (2012). Periostin promotes fibrosis and predicts progression in patients with idiopathic pulmonary fibrosis. Am. J. Physiol. Lung Cell Mol. Physiol..

[24] Okamoto M., Hoshino T., Kitasato Y., Sakazaki Y., Kawayama T., Fujimoto K., Ohshima K., Shiraishi H., Uchida M., Ono J. (2011). Periostin, a matrix protein, is a novel biomarker for idiopathic interstitial pneumonias. Eur. Respir. J..

[25] Guirao-Abad J.P., Shearer S.M., Bowden J., Kasprovic D.A., Grisham C., Ozdemir M., Tranter M., Wang Y., Askew D.S., Kanisicak O. (2024). Pulmonary fibroblast activation during. bioRxiv.

[26] Zhuang R., Chen J., Cheng H.S., Assa C., Jamaiyar A., Pandey A.K., Pérez-Cremades D., Zhang B., Tzani A., Khyrul Wara A. (2022). Perivascular Fibrosis Is Mediated by a KLF10-IL-9 Signaling Axis in CD4+ T Cells. Circ. Res..

[27] Wang J., Chen L., Chen B., Meliton A., Liu S.Q., Shi Y., Liu T., Deb D.K., Solway J., Li Y.C. (2015). Chronic Activation of the Renin-Angiotensin System Induces Lung Fibrosis. Sci. Rep..

[28] Chistiakov D.A., Killingsworth M.C., Myasoedova V.A., Orekhov A.N., Bobryshev Y.V. (2017). CD68/macrosialin: Not just a histochemical marker. Lab. Investig..

[29] Barhoumi T., Todryk S. (2023). Role of monocytes/macrophages in renin-angiotensin system-induced hypertension and end organ damage. Front. Physiol..

[30] Kang X., Zhao K., Huang Z., Fukada S.I., Qi X.W., Miao H. (2025). Pdgfrα. Genes Dis..

[31] Li R., Hanna A., Huang S., Hernandez S.C., Tuleta I., Kubota A., Humeres C., Chen B., Liu Y., Zheng D. (2024). Macrophages in the infarcted heart acquire a fibrogenic phenotype, expressing matricellular proteins, but do not undergo fibroblast conversion. J. Mol. Cell. Cardiol..

[32] Kasprovic D.A., Jaggers R.M., Tranter M., Kanisicak O. (2024). Cardiac macrophages and fibroblasts: A synergistic partnership without cellular transition. J. Mol. Cell. Cardiol..

[33] Gao F., Bai R., Qin W., Liang B., Yang Z., Yang H. (2022). Angiotensin II induces the expression of periostin to promote foam cell formation in oxLDL-treated macrophages. Int. J. Cardiol..

[34] Lerman L.O., Kurtz T.W., Touyz R.M., Ellison D.H., Chade A.R., Crowley S.D., Mattson D.L., Mullins J.J., Osborn J., Eirin A. (2019). Animal Models of Hypertension: A Scientific Statement From the American Heart Association. Hypertension.

[35] Biernacka A., Dobaczewski M., Frangogiannis N.G. (2011). TGF-β signaling in fibrosis. Growth Factors.

[36] Young O.N., Bourke J.E., Widdop R.E. (2023). Catch your breath: The protective role of the angiotensin AT. Biochem. Pharmacol..

[37] Wang T.N., Chen X., Li R., Gao B., Mohammed-Ali Z., Lu C., Yum V., Dickhout J.G., Krepinsky J.C. (2015). SREBP-1 Mediates Angiotensin II-Induced TGF-β1 Upregulation and Glomerular Fibrosis. J. Am. Soc. Nephrol..

[38] Sun Y., Zhang J., Zhang J.Q., Ramires F.J. (2000). Local angiotensin II and transforming growth factor-beta1 in renal fibrosis of rats. Hypertension.

[39] Wang Z., An J., Zhu D., Chen H., Lin A., Kang J., Liu W., Kang X. (2022). Periostin: An emerging activator of multiple signaling pathways. J. Cell Commun. Signal..

[40] Nanri Y., Nunomura S., Terasaki Y., Yoshihara T., Hirano Y., Yokosaki Y., Yamaguchi Y., Feghali-Bostwick C., Ajito K., Murakami S. (2020). Cross-Talk between Transforming Growth Factor-β and Periostin Can Be Targeted for Pulmonary Fibrosis. Am. J. Respir. Cell Mol. Biol..

[41] Davis J., Burr A.R., Davis G.F., Birnbaumer L., Molkentin J.D. (2012). A TRPC6-dependent pathway for myofibroblast transdifferentiation and wound healing in vivo. Dev. Cell.

[42] Uhal B.D., Kim J.K., Li X., Molina-Molina M. (2007). Angiotensin-TGF-beta 1 crosstalk in human idiopathic pulmonary fibrosis: Autocrine mechanisms in myofibroblasts and macrophages. Curr. Pharm. Des..

