# Supplementary material for: Angiotensin II Promotes Progressive Activation of Fibrogenic Periostin-Lineage Cells in Lung and Kidney
Source: Cells. 2025 Oct 11;14(20):1584. doi: 10.3390/cells14201584 (PMC12563049; doi:10.3390/cells14201584)
Supplement: Supplementary file 1 [file cells-14-01584-s001.zip › cells-3862025-supplementary.pdf]

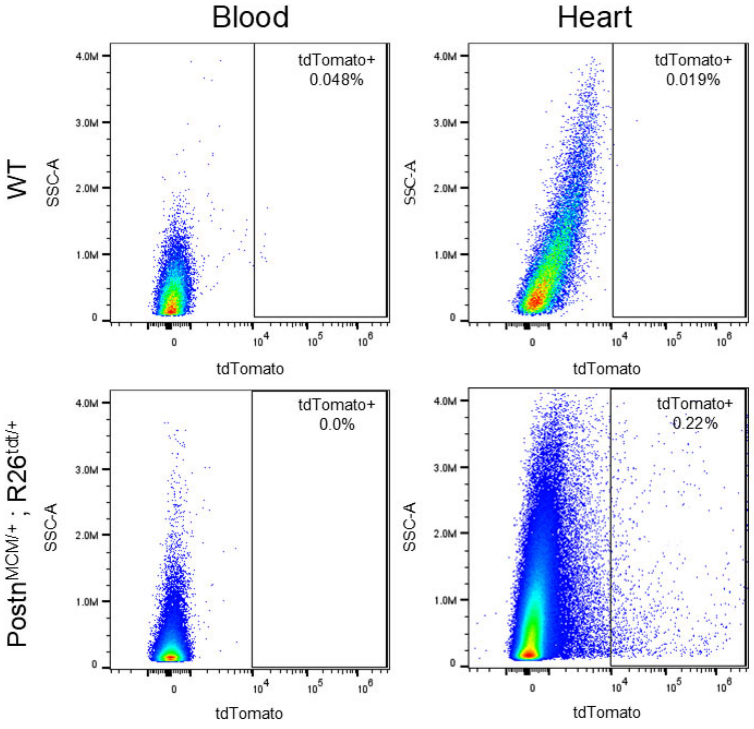

Supplemental Figure S1. Figure S1. Representative flow cytometry plots of isolated tdTom^{+} cells from blood and heart of AngII-treated wild-type (WT, n = 1) mice and Postn^{Lin} (Postn$^{MCM/+}$; R26$^{tdt/+}$) mice (n = 1).
